# Contested or complementary healing paradigms? Women’s narratives of COVID-19 remedies in Mwanza, Tanzania

**DOI:** 10.1186/s13002-021-00457-w

**Published:** 2021-04-26

**Authors:** Gerry Mshana, Zaina Mchome, Diana Aloyce, Esther Peter, Saidi Kapiga, Heidi Stöckl

**Affiliations:** 1 National Institute for Medical Research, Mwanza, Tanzania; 2 Mwanza Intervention Trials Unit, Mwanza, Tanzania; 3 London School of Hygiene and Tropical Medicine, London, UK

**Keywords:** COVID-19, SARS-CoV-2, Biomedicine, Traditional medicine, Religious healing, Women, Africa

## Abstract

**Background:**

COVID-19 has caused worldwide fear and uncertainty. Historically, the biomedical disease paradigm established its dominance in tackling emerging infectious illnesses mainly due to innovation in medication and advances in technology. Traditional and religious remedies have emerged as plausible options for prevention and treatment of COVID-19, especially in Africa and Asia. The appeal of religious and traditional therapies against COVID-19 in the African setting must be understood within the historical, social, and political context. This study explored how women and community members dealt with suspected symptoms of COVID-19 in Mwanza, Tanzania.

**Methods:**

This study was conducted in Nyamagana and Ilemela districts of Mwanza, Tanzania, between July and August 2020. We conducted 18 mobile phone in-depth interviews with a purposively selected sample of women aged 27–57 years participating in an existing longitudinal study. For safety reasons, smart mobile phones were used to collect the data. Each interview was audio recorded after obtaining verbal consent from the participants. The audio files were transferred to computers for analysis. Four researchers conducted a multistage, inductive analysis of the data.

**Results:**

Participants reported wide use and perceived high efficacy of traditional remedies and prayer to prevent and treat suspected symptoms of COVID-19. Use was either alone or combined with public health recommendations such as hand washing and crowd avoidance. Despite acknowledging that a pathogen causes COVID-19, participants attested to the relevance and power of traditional herbal medication and prayer to curb COVID-19. Four main factors underline the symbolic efficacy of the traditional and religious treatment paradigms: personal, communal, and official reinforcement of their efficacy; connection to local knowledge and belief systems; the failure of biomedicine to offer a quick and effective solution; and availability.

**Conclusions:**

In the context of emerging contagious illnesses, communities turn to resilient and trusted treatment paradigms to quell fear and embrace hope. To tackle emerging infections effectively, it is essential to engage the broader sociopolitical landscape, including communal considerations of therapeutic efficacy.

## Background

### Fear and uncertainty

The onset of SARS-CoV-2, the virus causing COVID-19 disease, created an atmosphere of fear and a sense of uncertainty all over the world. The outbreak was first reported in China in December 2019, and by March 2020, the World Health Organization (WHO) declared it a global pandemic [1]. The WHO makes frequent briefings about the rapid spread of the infections and mortality through its information networks and mainstream media. Likewise, governments provide country-specific updates on the number of cases and control measures, including testing. The main approach to control COVID-19 has been by imposing restrictions on social interactions and movement—commonly referred to as lockdown. Countries have implemented a variety of lockdown measures, ranging from total or complete lockdown, to partial lockdowns limiting some of the social interactions. Tanzania reported the first case of COVID-19 in March 2020 [2]. By May 2020, the official number of cases and deaths reached 509 and 21 respectively [3]. To curb the pandemic, the government took several measures including closing of educational institutions, banning large gatherings, and promoting hand washing with soap and social distancing.

Mainstream and social media reports have compared it with historical pandemics—such as the bubonic plague and the human immunodeficiency virus and acquired immunodeficiency syndrome (HIV/AIDS). It was predicted that the pandemic would have a devastating impact in the developing countries with weak health systems and lack of resources to mount an effective response. The worst was forecast about Africa, where it was anticipated that the pandemic would combine with other epidemic conditions (such as HIV/AIDS) to cause unprecedented health challenges [4, 5]. This assessment was based on the poor healthcare systems and infrastructure on the continent—specifically the inadequate number of hospitals, healthcare personnel, ventilators, and personal protection equipment (PPEs) to cater for the population [6].

Against this backdrop of fear and uncertainty, reports of the use of local remedies against COVID-19 emerged from different parts of the world, especially Africa and Asia [4, 6–8]. One of the commonest local remedies reported during the early phases of the pandemic in Africa was the artemisinin-based herbal drink in Madagascar. Religious prayers for prevention and relief from the illness were also reported [9]. In Tanzania, designated days for national prayers were announced. The use of traditional remedies and prayer against COVID-19 generated ongoing discussion about their effectiveness, since they have not undergone the required biomedical processes to ascertain their efficacy.

Uncertainty makes treatment-seeking behavior more complex. It sets into motion complex treatment-seeking choices and processes as communities use different means to ascertain the trustworthy of medications and their efficacy [10, 11]. To quell fear and embrace hope against new illness, communities tend to explore their sociocultural and local knowledge systems for answers [12–14]. This includes revisiting customary knowledge deep-rooted in the local sociocultural practices and belief systems. Traditional and religious illness paradigms (or models) offer social stability in the context of such widespread uncertainty. This was the case in the initial outbreaks of Ebola in East and West Africa [15, 16].

### Rival complementarity: biomedicine and religious and traditional healing paradigms in Africa

The interaction between different illness and healing paradigms is as old as history itself. This is evident through the accounts of how people from different parts of the world mingled for economic, social, or political reasons. The trade in spices for medicinal use is a good example. The import from Asia and establishment of clove (*Syzygium aromaticum* (L.) (Merr. et L.M.Perry)) plantations in East Africa for medicinal use (among other uses) are well recognized [17]. These interactions—whether peaceful or confrontational—set in motion the interface, complementarity, and ensuing contest for supremacy between illness paradigms originating in different societies. Illness paradigms are part of the cultural exchange between societies as they are an important social fabric rooted in the belief systems and worldviews [13, 14].

In Africa, colonialism and the spread of Christianity and Islam intensified the interactions between illness paradigms [18, 19]. However, the colonization and dominance of Christianity in large parts of the continent gave advantage to the biomedical disease model [19, 20]. The establishment of modern education and healthcare systems—mostly through missionary schools and hospitals—reinforced the dominance of the biomedical model. Medical education, research, and policy in the colonies further cemented the dominance, which continued into the post-colonial period [20]. Technological advancement and innovation on the diagnosis and treatment of disease in the western countries increased the supremacy of the biomedical disease model, not only in Africa, but worldwide [14, 20, 21]. It is important to acknowledge the contribution of biomedicine in reducing mortality and extending life expectancy in Africa and other developing countries. Equally, it is essential to recognize the role that other illness paradigms play in alleviating illness in these contexts.

Despite the historical dominance of the biomedical disease model, the traditional and religious illness paradigms persist in Africa and other parts of the world. The resilience of the traditional paradigm in the African setting despite large sections of the population receiving modern education and conversion to Christianity and Islam illustrates the significance of cultural knowledge and beliefs to social identity and wellbeing [14, 19]. As an adaptive or coping mechanism, many African communities embrace medical pluralism either concurrently or serially [12, 22, 23]. Through medical pluralism, the traditional and religious treatment paradigms remain relevant and resilient despite increasing modernization.

In Africa, treatment of a wide range of communicable and non-communicable illnesses through local remedies and prayer is well documented [12, 14, 19, 22]. Of recent, these paradigms have been applied against outbreaks of infectious illnesses such as malaria, HIV/AIDS, and Ebola [15, 16, 23–25]. Thus, the appeal of religious and traditional remedies against COVID-19 must be understood within this historical, social, and political context. In this paper, we use the term traditional medicine and local remedies interchangeably to refer to a wide range of herbal remedies in their various forms. This does not imply that these remedies originate in these communities, but as already shown, historically, there is exchange and adaptation of treatment paradigms between societies. We use the term religious healing to refer to religious- or spiritual-based practices against illness. These are grounded on the wide range of religious beliefs in Tanzania, but predominantly Christian and Islam.

### Symbolic efficacy and power

Social scientists have highlighted the complexity of how societies around the world determine and perceive treatment efficacy, apart from just the physiological and clinical evidence. Claude Levi-Strauss, a renowned anthropologist, coined the concept “symbolic efficacy” to explain how beliefs generate cure from sickness [26]. He studied how in different societies, rituals and magical symbols led to relief and cure from illness. Strauss was intrigued by how the “mere” words of a shaman (healer) drew on multifaceted cultural belief systems to produce cure from illness.

Symbolic efficacy connects to community knowledge and belief systems about the appropriate ways of tackling illness [21]. These prescriptions become “reality” and are readily embraced in society [27]. Beliefs in the efficacy of traditional healing practices are socially constructed and maintained [26]. Bourdieu [27] argues that the power of symbolic efficacy depends on the degree upon which the proposed declaration is founded in reality. Hence, communal declaration of the efficacy of a particular remedy against a specific disease gains traction if it connects to the local and socially maintained knowledge and worldviews. Communal inquiry, recognition, and comparison of the symptoms of a new illness with those of a known one—including how it is treated—create a “reality” of the ability of the remedies to contain it. Symbolic efficacy is thus established on the sociocultural foundations of communal knowledge and world views, giving it a seal of trustworthiness to individuals and the wider community.

Symbolic efficacy is closely connected to symbolic power. Symbolic power is the ability to constitute reality and establish the appeal and trustworthiness of a declaration [27]. In this case, the official and communal declarations on the efficacy of religious and local remedies against COVID-19 matter. People are more likely to accept health recommendation from a source within their close social networks or those in authority. Such declarations carry tremendous symbolic power of appeal to specific communities, despite contra pronouncements by the global public health actors such as the WHO. Therefore, the interpretation of the wide use and appeal of religious and local remedies against COVID-19 in Africa and other settings require an in-depth analysis which gives due attention to the sociocultural and policy context. This paper argues that in order to tackle emerging infectious illnesses across different settings, public health responses need to engage traditional and religious models, including the imbedded social constructs of therapeutic efficacy.

## Methods

### Study design and sampling

**Fig. 1:**
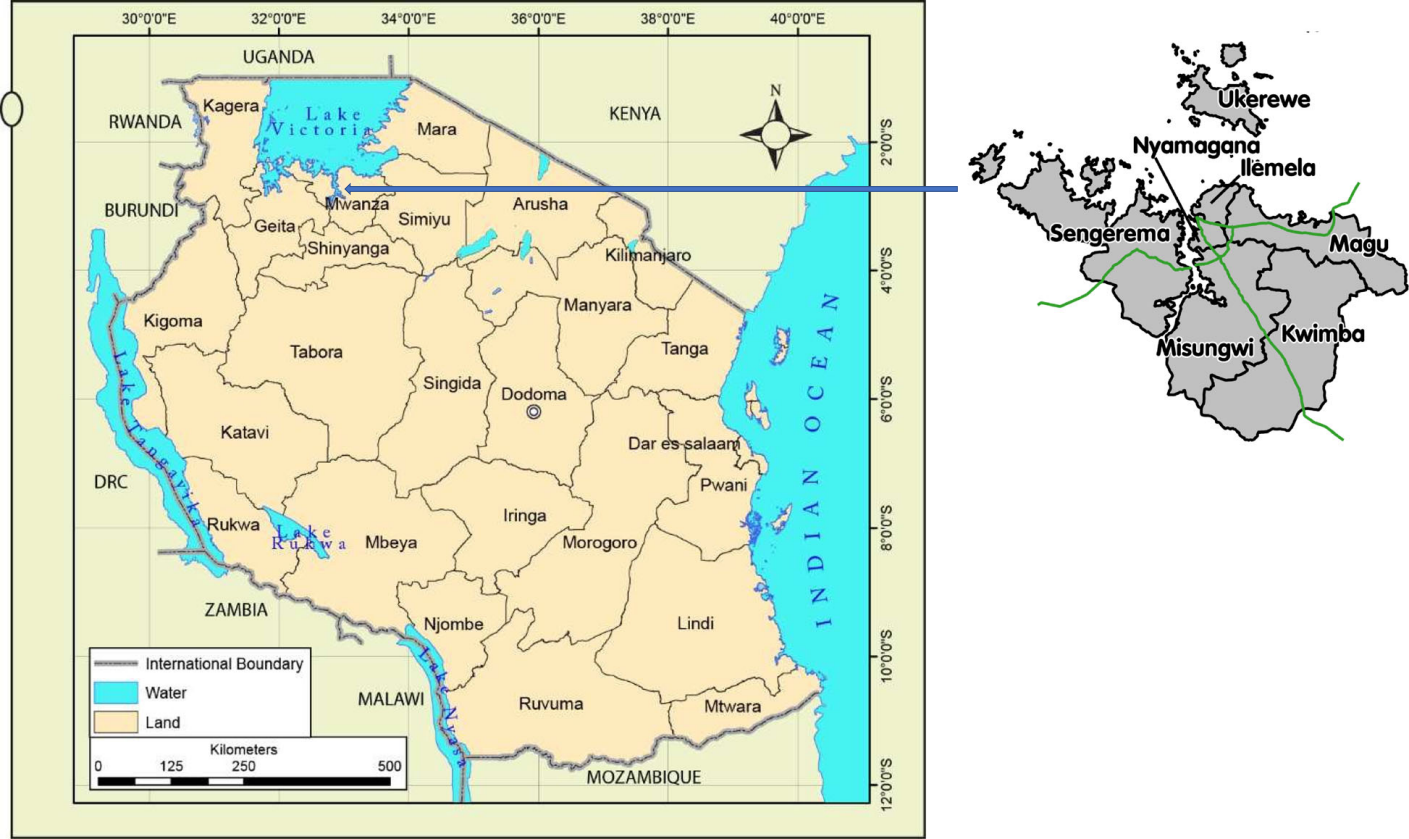
Map of Tanzania showing Nyamagana and Ilemela Districts in Mwanza region

This paper draws from mobile phone in-depth interviews with 18 purposively sampled women participating in the MAISHA longitudinal study in Mwanza city, Tanzania. The study was carried out in Nyamagana and Ilemela districts which constitute Mwanza city (Fig. 1). Mwanza is the second most populous city in the country located on the southern shores of Lake Victoria. The MAISHA study investigates the predictors and consequences of intimate partner violence [28]. The age of the women was between 27 and 57 years. The participants were purposively sampled to capture the diverse sociodemographic characteristics of other women in Mwanza, such as age, nature of income-generating activities, religion, and the status of relationship with their male partners (Table 1). After the outbreak of COVID-19 in Tanzania, the Medical Research Coordination Committee suspended most of the research activities in the country for safety reasons. The MAISHA study investigators decided to suspend all face to face fieldwork activities, and instead designed a low risk, phone-based nested study to explore the effects of COVID-19 to the women enrolled in the study. The mixed methods study included components of a phone survey and qualitative interviews. This paper draws on the qualitative phone interviews as they were rich in detail about how the women and communities dealt with the threat of COVID-19, including the treatment practices. The interviews provided detailed insights about social interactions and communal beliefs about the illness. Since none of the participants or family members had a confirmed COVID-19 diagnosis, we refer to *COVID-19-like symptoms* when respondents talked about episodes they linked to the illness.

**Table 1:** Participant demographic Information

Age	Marital status	Education	Occupation	Religion	Ethnic group
43	Married	Completed secondary school	Tailor	Christian	Ngoni
45	Married	Completed primary school	Farmer	Christian	Sukuma
44	Married	Completed secondary school	Farmer	Adventist	Sukuma
48	Widow	Incomplete primary school (Std 2)	Florist	Muslim	Haya
43	Married	Incomplete secondary school (Form 2)	Unemployed	Muslim	Pare
32	Divorced	Completed primary school	Unemployed	Christian	Jita
37	Married	Completed primary school	Entrepreneur	Christian	Nyakyusa
27	Single	Diploma	Entrepreneur	Christian	Sukuma
45	Married	Completed primary school	Entrepreneur	Christian	Ngoni
37	Divorced	Incomplete primary school (Std 2)	Unemployed	Christian	Sukuma
45	Married	Completed primary school	Entrepreneur	Christian	Haya
30	Married	Completed primary school	Entrepreneur	Christian	Nyambo
57	Married	Completed primary school	Entrepreneur	Christian	Sukuma
36	Divorce	Diploma	Hotelier	Christian	Sukuma
43	Married	Completed primary school	Entrepreneur	Christian	Hangaza
41	Married	Completed primary school	Entrepreneur	Muslim	Haya
49	Married	Completed primary school	Unemployed	Christian	Sukuma
43	Married	Completed primary school	Entrepreneur	Muslim	Sukuma

### Data collection

Two female graduate interviewers were trained to conduct the mobile phone interviews. The two researchers had previously conducted face to face in-depth interviews with the women exploring intimate partner violence. Thus, the COVID-19 mobile phone qualitative interviews benefited from established rapport and trust from the previous interactions. As such, the participants were happy when contacted by the same interviewers to join in the COVID-19 study. The team had already obtained the phone numbers of the participants from previous contact for the MAISHA study.

The interviewers received extensive 1-week training incorporating theoretical and practical sessions. The training considered the challenges of conducting phone-based interviews in the developing country setting such as Tanzania—such as poor phone network. The practical sessions included mock interviews with other staff—to assess the appropriateness of the questions and length of the interviews. It was important to ensure that the interviews were not lengthy in respect of the respondent’s time and to avoid interruptions from poor phone network and low battery charge. The interviews were conducted in Swahili (national language) and took approximately between half an hour and 1 h and 20 min. Participants were reimbursed a total of 8000 Tanzanian shillings (about 3.5 US dollars) through their mobile phones to compensate for their time.

### Ethical considerations

At first, the interviewers called the participants to inform them about the study and obtain their consent. Thereafter, they set appointments for the phone interviews at the time suitable for the participants. To maintain confidentiality and avoid interruptions from other staff, the researchers conducted the interviews in dedicated rooms in the office. The study received ethics approval from the Tanzanian Medical Research Coordination Committee (NIMR/HQ/R.8c/Vol. I/837) and the Ethics Committee of the London School of Hygiene and Tropical Medicine (Ref: 11918-3).

### Data analysis

Smartphones with audio-recording capacity were used for the interviews. All interviews were audio recorded after obtaining permission from the participants. After each interview, the audio files were transferred to secured computers in the office for analysis. The analysis was in three main stages using the audio files in original language (Swahili) as the primary source. Swahili is the first language of the four authors involved in the analysis and interpretation (GM, ZM, DA, and EP).

In the first stage of data analysis, the two interviewers wrote a detailed summary of each interview in English, soon after completion. The summaries—covering a wide range of topics—followed an outline reviewed by five authors of this paper (GM, ZM, DA, EP, and HS). Subsequently, the two interviewers listened to all the audio recordings of the interviews to enrich their summaries. These summaries identified the main themes emerging from the data. In the second stage, two senior social scientists (GM and ZM) examined the summary notes and the emerging themes, and listened to the audio recordings to appraise them. Analytic notes from the second stage of the data review were made, compared, and discussed to appraise the emerging concepts and themes. In the final stage, an interactive process of theorizing and data appraisal (led by GM) was done before the findings were written up.

After completion of the interviews and analysis of data, one of the authors (DA) worked with a consultant botanist to identify and collect specimen vouchers of all the plants reported to be used for prevention and treatment of suspected COVID-19 symptoms. Some of the specimens were collected from homes of the research team members, and the others were purchased from a local market in Mwanza city. We have reported the scientific and family names of all the plants according to the Plants of the World Online (POWO) recommendation.

## Results and discussion

### Medical pluralism for prevention and treatment

Studies have shown that beliefs in the multiple causes of disease informs the use of a mix of biomedical, traditional, and religious treatment paradigms—either serially or concurrently [12, 14, 22]. However, the majority of our respondents attributed the cause of COVID-19 to a pathogen. A few were specific that it is caused by a virus. There were no reports of beliefs in supernatural causes.

Although our study participants attributed COVID-19 to a biological cause, traditional remedies and prayer were reported to be used for protection and treatment against suspected COVID-19 symptoms. In contrast, other studies in Tanzania have reported use of traditional remedies or prayers only when the cause was attributed to supernatural causes [22, 24]. In this study, remedies against COVID-19 symptoms were mostly used in combination with the recommended public health protection measures, mainly hand washing and social distancing. A few reported the use of hand sanitizers and facial coverings (masks). These recommended public health protection measures were used in the context where traditional remedies and prayer were extensively practiced. In-depth analysis of the narratives revealed not only the use of these paradigms against suspected COVID-19 symptoms, but also strong conviction about their effectiveness.

I ate natural foods like fruits, vegetables also ginger, cloves and other spices. When you use them often you are safe. Also, by steaming myself at least three or two times in a week by mixing lemon grass and other ingredients. I also washed my hands well, carried my mask when I wanted to go out. I also took my sanitizer. [IDI 07, 44 years, shop owner, Christian].

I had a neighbor who almost had all the symptoms of the disease. People were not even getting close to greet her so she had to lock herself up. She stayed inside for about a month. I remember I went to greet her and came back home very worried. I was very worried and I steamed myself with local herbs concentration upon my arrival at home. I also took the Alur tablets. Her family wasn’t allowed to visit other people either, so it was like they had their own lockdown. They steamed with local herbs, used orange juice and sanitizers to protect themselves. They protected themselves to protect others. [IDI 01, 44 years, Christian, small scale trader and street leader].

### Confronting the threat and symptoms: traditional remedies for prevention and treatment

The participant’s narratives showed that there was widespread use of a combination of local herbs, common tree leaves, and vegetables for the protection against suspected COVID-19 symptoms. From the interviews, a total of 19 plants were reported to be used against suspected COVID-19 symptoms (Table 2). These included leaves of several trees such as eucalyptus (*Eucalyptus globulus* Labill.), lemon (*Citrus limon* L.), neem (*Azadirachta indica*. A.Juss), and guavas (*Psidium guajava* L.). Also, leaves of plants such as lemon grass (*Cymbopogon citratus* Stapf*.*), *Kashwagara/Malumbalumba* (*Ocimum gratissimum* L.), and *mabingobingo* (*Pennisetum purpureum* Schumach.) and other local herbs such as *mlavumba* (*Tetradenia riparia* (Hochst.)). Ginger (*Zingiber officinale* Roscoe), red onions (*Allium cepa* L.), garlic (*Allium sativum* L.), and lemons were also used in preparation of the remedies. Honey was also reported to be used in the preparation of the remedies. The concoctions were used in two main ways: either crushed and mixed with warm water or boiled in water and the product taken orally. Alternatively, the concoctions were boiled and the steam inhaled through the mouth and nose.

**Table 2:** Plants used for prevention and treatment of suspected COVID-19 symptoms

Plant name	Family	Swahili name	Local/ethnic name	English name^**1**^	Part used	Voucher No.
*Allium cepa* L.	Amaryllidaceae	Kitunguu maji		Red onion	Bulb	DA 15/21
*Allium sativum* L.	Amaryllidaceae	Kitunguu swaumu		Garlic	Bulb	DA 14/21
*Azadirachta indica*. A.Juss	Meliaceae	Muarobaini		Neem tree	Leaves	DA 04/21
*Capsicum frutescens* L.	Solanaceae	Pilipili kichaa		Chilli pepper	Fruits	DA 16/21
*Carica papaya* L.	Caricaceae	Mpapai		Papaya	Leaves	DA 06/21
Citrus limon L.	Rutaceae	Mlimao		Lemon	Fruit	DA 17/21
*Citrus sinensis* L.	Rutaceae	Mchungwa		Orange	Fruit	DA 18/21
*Cymbopogon citratus* Stapf.	Poaceae	Mchaichai		Lemon grass	Leaves	DA 09/21
*Eucalyptus globulus* Labill.	Myrtaceae	Mkaratusi	-	Eucalyptus	Leaves	DA 01/21
*Mangifera indica* L.	Anacardiaceae	Muembe		Mango	Leaves	DA 05/21
*Ocimum canum* L.	Lamiaceae	Kivumbasi/Mvunja homa		-	Leaves	DA 03/21
Ocimum gratissimum L.	Lamiaceae	***-***	-Kashwagara (*Haya*) -Malumbalumba (Sukuma/Nyamwezi)	African basil	Leaves	DA 02/21
*Pennisetum purpureum* Schumach.	Poaceae	Mabingobingo	Mabingobingo (Sukuma)	Elephant grass	Leaves	DA 10/21
*Persea americana* Mill.	Lauraceae	Mparachichi		Avocado	Leaves	DA 07/21
*Psidium guajava* L.	Myrtaceae	Mpera		Guava	Leaves	DA 08/21
*Ricinus communis* L.	Euphorbiaceae	Mnyonyo		Castor	Leaves	DA 19/21
*Syzygium aromaticum* (L.) (Merr. et L.M.Perry)	Myrtaceae	Karafuu		Cloves	Dried flower buds	DA 13/21
*Tetradenia riparia* (Hochst.)	Lamiaceae	-	Mlavumba (*kiha*)	-	Leaves	DA 11/21
*Zingiber officinale* Roscoe	Zingiberaceae	Tangawizi		Ginger	Roots	DA 12/21

^1^The authors identified the English names which were then confirmed using the Plants of the World (POWO) website and google search engine.

From the interviews, inhaling the steam (*kujifukiza* or *kupiga nyungu* in Swahili) was the most popular way of administering the concoctions. A mixture of the tree leaves and local herbs was boiled in a huge cooking pot. At the point of boiling, the pot was taken off the cooker, and the covering lid removed. Then, the person needing treatment sat next to the pot and was covered with a bed sheet, blanket, or any other large piece of cloth to ensure that none of the medicinal steam escapes. They would inhale the steam and sweat for some time (for some, up to about 30 min). The sweating indicated that the medicine has entered into the body and taken the illness out of it. Respondents who steamed reported feeling relief and improvement after the sessions.

On my side, I have steamed myself a lot, my kids as well did that. They also washed their hands and applied sanitizers, we did that often. But what I know about steaming process is that you really sweat. The ingredients that I used were eucalyptus, guava leaves and other herbs. We boiled them then steamed ourselves using a sheet that doesn’t allow air to penetrate, we all did this, even my husband. After the whole process we felt okay, you feel like you’re no longer tired…we decided to steam ourselves because we heard people saying that the disease has no cure so we should take all the necessary precautions and even steam ourselves. [IDI 02, 44 years, small scale business -selling undergarments-, Christian (SDA)].

I mean you take the natural herbs like ‘kashwagara’, eucalyptus, neem, cut pieces of lemon, blend ginger then boil the mixture in a big pot. You find a very heavy sheet which you use to cover yourself…when the mixture boils, you cover yourself and stir the mixture using a stick or wooden spoon, the vapor penetrates all parts of your body especially the mouth and nose. This will kill the virus and prevent corona because you will sweat very much and corona doesn’t live in a warm or hot environment…What else works best rather than steaming my dear? [ IDI 04, 48 years, Florist, Muslim]

Oral remedies were consumed immediately, and the remainder consumed later in the day or in subsequent days. The use of traditional remedies was mainly for home-based treatment. Adults would prepare the remedies and use them themselves or give their family members. The leaves of herbs were usually obtained free of charge either from their homesteads, a neighbor’s home, or from a tree or plant in a public space. The vegetables (such as ginger, lemons, and onions) were purchased from markets as part of the normal household shopping. From the interviews, there was no clarity about the exact formula for mixing the different ingredients or the specific mixture of ingredients which was most effective.

There is this mixture of a lemon, 4 hot chili peppers, ginger a size of a finger, and 10 kernel pieces of a garlic onion. These are all mixed with 1 liter of water. Or if you have a blender you may blend them. You drink two food spoons thrice per day. This mixture is used for drinking not steaming. [IDI 03, 49 years, Christian, small scale trader].

The same participant continued with her narrative later in the interview:

A few used the traditional ways of steaming with traditional herbs... I would meet a few people in search of the leaves I told you were mixed for that. Some used the papaya leaves, lemongrass, neem leaves and others.

Personal experience is vital for establishing symbolic efficacy [26]. Trustworthiness of the efficacy of herbal steaming and use of other remedies was drawn from personal experience of its effectiveness against other illnesses. Local knowledge and memory about the effectiveness of specific remedies in curing previous illness episodes helped to validate their perceived efficacy [12, 14, 22]. Efficacy was further reinforced through the testimonies of those in their close social networks—such as family and friends. Personal experience of the relief brought by steaming and similar observations of family members verified the perceived effectiveness of the remedies. Consequently, these experiences and communal narratives reinforced belief about their efficacy.

So, what I do is use the lemon grass and other things like neem, mango leaves, avocado leaves and lemons, so I mixed them all together. As I said they are all medicines. Let me give you explanation on guava leaf. Even before corona they were used to cure diseases like when a person had diarrhea, so what you do is squeeze the leaves or boil the leaves then drink the water from the leaves it really helps, you will stop diarrhea. So you can just imagine if it cures that in the stomach of a person it means it’s likely to help even in killing virus like corona. So, for me I truly believe in such herbs that they can even treat corona. [IDI 07, 44 years, shop owner, Christian].

All my neighbors are from an ethnic group known as Haya. Steaming themselves with local herbs is a common practice even before this problem started. Even when they just felt a little ill, they would mix different leaves and steam themselves for three consecutive days then tell you they no longer have Malaria. So, they steamed a lot during this period. [IDI 09, 45 years, small scale business, Christian].

### Divine intervention: prayer for protection

Study participants described that praying was effective for the protection against COVID-19. In particular, they outlined three types of prayers: *prayer for self*, *family*, and *country*. These categories of prayers demonstrate the social obligation of individuals and community members to confront the illness. Furthermore, the comprehensive prayers—covering the entire social spectrum—show communal obligation to act against the illness [9]. In this setting, illness requires a broader social response rather than it being just the concern of an individual or immediate family [12, 22]. The following excerpts illustrate these points:

I prayed that this problem should not affect my children. I hoped that I don’t lose any of my relatives especially when I heard the disease was increasing in Dar es Salam…because three quarters of my relatives live there. This took away my peace of mind but I am thankful to God that they are all doing fine and no one got the illness. [IDI 09, 45 years, Christian, small scale trader]

We prayed and reminded God to protect Tanzania from the corona virus disease and he listened. The situation is as you can see right now. We don’t wear masks like before. We only wash our hands with flowing water, we stopped [mass] gathering, but now we can attend our gatherings as usual. [IDI 01, 44 years, Christian, small scale trader and street leader].

As the two excerpts illustrate, the respondents were confident that prayer was efficacious for the protection against COVID-19. To them, it was a sure way for protecting themselves, their families, and the nation as a whole. There was no doubt in their minds on its effectiveness as none of their family members were infected. Likewise, they were convinced that the threat against their communities and nation was removed—evident through the discontinued use of the public health protection measures, such as hand washing and avoidance of large gatherings.

In some contexts, prayer enhances communal and national cohesion in times of difficulties [9, 19]. Having a national day of prayer irrespective of the variety of religions and beliefs in a country displays social solidarity. Joint prayers convey a sense of consensus and a common societal approach to overcoming the pandemic—without monopoly of one religion. It also enhances hopefulness by bringing different sections of the communities together—including those with antagonistic beliefs—to jointly face the uncertainty from the pandemic.

We just left other things to God. He is the one who protected us from this disease because we are still here until now while we hear other people dying of the disease frequently. The main protection is through praying to God. [IDI 15, 43 years, Christian, small scale trader]

Prayer was used either exclusively or in combination with other paradigms. This has been noted in other African countries—for example when dealing with the uncertainty brought by infectious illnesses like HIV/AIDS [25, 29]. In this study, prayer was either used in combination with public health prevention measures—mostly hand washing, or with traditional herbs. Some participants believed that prayer alone was sufficient, thus not requiring any other additional interventions. However, most of the participants believed it should be used in combination with the public health interventions and traditional remedies:

People used the steaming method, but for me I didn’t steam myself at all, I just prayed to God for protection, washed my hands often because you can find yourself holding things as you interact with people… [IDI 08, 37 years, Christian, casual laborer].

For me I believe praying works and it’s the best decision by praying to our God and also continue taking the necessary precautions like social distancing, wearing of masks and other precautions... [IDI 10, 45 years, Christian, charcoal trader]

### Drawing on symbolic power: communal and official approval

Communal and official declarations of the efficacy of traditional and religious remedies against COVID-19 created “reality” to the study participants, their families, and the wider community. Personal narratives of relief from the threat and COVID-19-like symptoms, observations of family members recovering, and testimonies from others in the community established the perceived efficacy of these interventions. Their trustworthiness and therapeutic efficacy [26] became reputable through personal experience, observation, and conformity with the local cultural and religious belief systems.

People used the traditional herbs as well, you would see them coming to my home telling me about a certain tree leaf they want around my house to go mix with other herbs then steam themselves…so we exchanges ideas on ingredients used for steaming. [IDI 02, 44 years, Christian (SDA) small scale trader].

I think the best one is the alternative method (njia mbadala – alternative way) of steaming with local herbs…because of the nature of the illness. It causes chest pain and rib cage pain and most people who used this alternative method of steaming with local herbs recovered. [IDI 05, 43 years, small scale trader, Muslim].

What I know about the traditional cure is that a lot of people were steaming with the local herbs. Even I with my family steamed with the local herbs concentrates…We used to boil herbs and steamed the vapour by covering ourselves with the bed sheets until when we had enough heat. [IDI 18, 44 years, small scale trader, Muslim].

The reality of these declarations was further recognized through connection to communal perspectives that dealing with illness is a social responsibility. This was evident through the social-wide prayers covering family, community, and nation. As already shown, some of the participants prepared traditional remedies for their family members—confirming the societal nature of these interventions. Social scientists have long argued for biomedicine to embrace the comprehensiveness of how different societies define and respond to illness, including how they explain therapeutic efficacy [12, 14, 20, 21].

I did nothing else apart from praying to God to protect my family and the whole community at large. [IDI 03, 49 years, Christian, small scale trader].

My eldest son who lives in Dar-es-Salaam, told me he felt his lungs were painful, he was also coughing and had flu, so he had to steam himself by using the ingredients. I told them about taking hot water which was mixed with lemons and ginger. They had to make the mixer really strong so that it can help him. He was sick for almost a week, but he later felt better. [IDI 16, 41 years, Muslim, second hand clothes trader].

It has been argued that power is the production of causal effect through social relations [30, 31]. Through symbolic power, the declarations of community and government officials encouraged the use of religious and traditional remedies against COVID-19. Such declarations made from positions of social or political authority were interpreted as “facts” by the population [27], thus encouraging specific treatment-seeking behaviors. Through symbolic power, the efficacy of these paradigms was accepted as they were made by those in charge of public health systems and policy. The appeal and impact of such pronouncements should never be underestimated, as they attract a substantial public response. Participants recounted how hearing these declarations encouraged them to pray and herbal steam against COVID-19-like symptoms:

Let me tell you something, when the disease outbreak looked like it was getting high, [mentions name of government official] insisted that people should start steaming themselves with local herbs (kujifukiza). The local leaders also started insisting on that, so people used the herbs a lot [IDI 01, 44 years, Christian, small scale trader and street leader].

Some of the respondents questioned the effectiveness of biomedicine against COVID-19 from what they heard through the media. This was in contrast to their firsthand experience and communal narratives about the effectiveness of traditional remedies and prayer. These remedies calmed the uncertainty of depending entirely on an elusive biomedical solution.

I don’t know about hospital treatments especially in Tanzania, but if medically treatment works why do people in western countries die!? Like we see death rates increasing daily, we listen and look at the news and see how the situation is in those countries that is really threatening, even in Kenya we hear people dying as well. [IDI 04, 48 years, Muslim, Florist].

I would opt for traditional medicine, because in the hospital there is no any medicine to cure the disease, so by boiling the traditional medicine and drinking it, kills the virus and cures you. So traditional medicine works best. [IDI 14, 37 years, unemployed, Christian].

## Conclusions

This study shows that the use of traditional remedies and prayer—alongside public health measures—against suspected COVID-19 symptoms is common in Tanzania. The symbolic efficacy of these paradigms was acknowledged through experience of personal relief from the threat and suspected COVID-19 symptoms. These were reinforced through observation and matching reports from those in the close social networks of the participants—especially family members. Accompanying testimonies about their efficacy from other community members and public officials strengthened their perceived efficacy. Since the remedies were mostly obtained at no cost or as part of family normal shopping, it made them desirable for use.

As recent survey reported wide use of food medicines for treatment of COVID-19 in different parts of the world [8]. In particular, there was increased consumption of ginger and garlic. The use of onion, turmeric, and lemon was also notably high. The findings from the survey and this study demonstrate how different communities around the world embrace food and traditional medicine in response to outbreaks of infectious diseases. Systematic studies on the use of food and traditional medicine against infectious diseases such as COVID-19 are needed, including robust analyses of their sociocultural context and appeal.

These findings underline the significance of social relations in treatment-seeking practices [12, 22, 26]. In the context of uncertainty from the COVID-19 pandemic, participants and community members adhered to the recommendations of those in their social sphere on how to deal with it. Trustworthiness of the remedies was reinforced through social recommendation [10, 11]. In this case, the power of social relations to influence health-seeking behavior is evident—through production of the recommended behavioral outcomes [30, 31]. Therefore, analysis and engagement of power relations should be an important component of community-based public health interventions.

A variety of reasons are given for the low morbidity and mortality from COVID-19 in Africa, in contrast to the initial predictions. These include the continent’s young population, pre-existing immunity, climatic variations, genetic factors, and behavioral differences [5]. The use of traditional remedies and prayer in combination with public health interventions such as hand washing helped to address hopelessness, and to give communities courage that they could avoid getting COVID-19, or recover and survive from it. Maintaining hope through religious beliefs and use of traditional medicine is shown to be beneficial to sufferers of other illnesses such as AIDS and cancer [32, 33]. These findings suggest that the psychosocial benefits of traditional remedies and prayer against emerging illnesses need further exploration.

The effectiveness of a comprehensive approach to tackling illness by addressing both—the physiological and psychological needs—is known, though not widely used [21]. Engaging local constructs of efficacy is crucial for the effective control of emerging illnesses in varied geographical settings [12, 19, 20]. Although there is no validation of the biological efficacy of these remedies against COVID-19, their use—together with public health means—require an in-depth inquiry rather than downplaying. Their role in creating communal and social stability amidst outbreaks of infectious illness is important.

The supportive policy environment for religious and traditional remedies in these settings suggest that they will continue to complement or even challenge the historical dominance of the biomedical disease model. Approval from community leaders and public officials indicate that they will remain important even when vaccines or biomedical cure for COVID-19 become available in these settings. In contexts where medical pluralism is widespread—such as in Africa—exploring their use and perceived efficacy should be part of the agenda for tackling COVID-19 and other emerging infectious illnesses.

This study has some limitations. First, there was no laboratory or clinical confirmation of COVID-19 infection from the symptoms reported by the respondents. Therefore, we refer to these reported episodes as *suspected COVID-19 symptoms*. Secondly these findings are based on interviews with 18 women in an urban city in North-western Tanzania. Nevertheless, the diverse sociodemographic characteristics of the participants such as their level of education, income-generating activities, religion, and ethnic groups provide rich insights into the variety of treatment practices for suspected COVID-19 symptoms in this context. Studies with larger sample sizes should explore further these treatment practices in this population or other similar contexts.

## Data Availability

Anonymized transcripts of the interviews are stored in secured computers and servers at the National Institute for Medical Research, Mwanza Centre. The transcripts are available upon request and subject to approval by the Tanzanian Medical Research Coordination Committee. Contact National Institute for Medical Research, 3 Barack Obama Drive, P.O.Box 9653, 11101 Dar-es-Salaam, Tanzania. Tel: +255 222121400, Email: ethics@nimr.or.tz

## References

[1] World Health Organization. WHO Director General’s opening remarks at the media briefing on COVID-19 – 11 March 2020 https://www.who.int/dg/speeches/detail/who-director-general-s-opening-remarks-at-the-media-briefing-on-covid-19%2D%2D-11-march-2020

[2] Tarimo CS, Wu J (2020). The first confirmed case of COVID-19 in Tanzania: recommendations based on lesson learned from China. Trop Med Health..

[3] World Health Organization. Coronavirus disease 2019 (COVID-19): situation report-128. 27 May 2020. Accessed June 22 2020.

[4] Dandara C, Dzobo K, Chirikure S. COVID-19 pandemic and Africa: from the situation in Zimbabwe to a case for precision herbal medicine. OMICS: A Journal of Integrative Biology. 2020; ahead of print 10.1089/omi.2020.0099 10.1089/omi.2020.0099 32654634

[5] Marsh K, Alobo M. COVID-19: examining theories for Africa’s low death rates. The Conversation, 7^th^ October 2020.

[6] Xu J, Zhang Y. Complementary therapies in clinical practice. 2020; 39, doi: 10.1016/j.ctcp.2020.101165, 101165 10.1016/j.ctcp.2020.101165 PMC7118627 32379692

[7] Mirzaie A, Halaji M, Dehkordi FS, Ranjbar R, Noorbazargan H A Narrative literature review on traditional medicine options for treatment of coronavirus disease. 2019 (COVID-19). Complement Ther Clin Pract. 2020; 40:101214.doi: 10.1016/j.ctcp.2020.101214. Epub 2020 Jun 17. PMID: 32891290. 10.1016/j.ctcp.2020.101214 PMC7831809 32891290

[8] Pieroni A, Vandebroek I, Prakofjewa J, Bussmann RW, Paniagua-Zambrana NY, Maroyi A, Torri L, Zocchi DM, Dam ATK, Khan SM, Ahmad H, Yeşil Y, Huish R, Pardo-de-Santayana M, Mocan A, Hu X, Boscolo O, Sõukand R (2020). Taming the pandemic? The importance of homemade plant-based foods and beverages as community responses to COVID-19. J Ethnobiol Ethnomed.

[9] Isiko AP (2020). Religious construction of disease: an exploratory appraisal of religious responses to the COVID-19 pandemic in Uganda. Journal of African Studies and Development..

[10] Hamill H, Hampshire K, Mariwah S, Amoako-Sakyi D, Kyei A, Castelli M (2019). Managing uncertainty in medicine quality in Ghana: the cognitive and affective basis of trust in a high-risk, low-regulation context. Soc. Sci. Med..

[11] Hampshire K, Hamill H, Mariwah Simon, Mwanga J, Amoako-Sakyi D. The application of Signalling Theory to health-related trust problems: the example of herbal clinics in Ghana and Tanzania. Soc. Sci. Med. 2017; 188:109-118, doi: 10.1016/j.socscimed.2017.07.009. Epub 2017 Jul 15. 10.1016/j.socscimed.2017.07.009 PMC5559643 28738317

[12] Carruth L (2014). Camel milk, amoxicillin, and a prayer: medical pluralism and medical humanitarian aid in the Somali Region of Ethiopia. Soc. Sci. Med..

[13] Mshana G, Plummer M, Wight D, Wamoyi J, Salamba Z, Ross D (2006). She was bewitched and caught an illness similar to AIDS: AIDS and sexually transmitted infection causation beliefs in rural Mwanza, Tanzania. Culture, Health and Sexuality..

[14] Sams K (2017). Engaging conceptions of identity in a context of medical pluralism: explaining treatment choices for everyday illness in Niger. Sociology of Health & Illness..

[15] Hewlett BS, Amola RP (2003). Cultural contexts of Ebola in Northern Uganda. Emerging Infectious Diseases..

[16] James PB, Wardle J, Steel A, Adams J (2019). Pattern of health care utilization and traditional and complementary medicine use among Ebola survivors in Sierra Leone. PLOS ONE..

[17] Martin PJ (1991). The Zanzibar Clove Industry. Economic Botany..

[18] Redkal OB (1999). Cross-cultural healing in East African ethnography. Medical Anthropology Quarterly..

[19] Sugishita K (2009). Traditional medicine, biomedicine and Christianity in Modern Zambia. Africa..

[20] Sindiga I (1995). African ethnomedicine and other medical systems, in Sindiga I, Nyaigotti-Chacha, Kanunah MP (edited) Traditional medicine in Africa.

[21] Moerman DE (1979). Anthropology of symbolic healing. Current Anthropology..

[22] Mshana G, Hampshire K, Panter-Brick C, Walker R (2008). Urban-rural contrasts in explanatory models and treatment-seeking behaviours for stroke in Tanzania. Journal of Biosocial Sciences..

[23] Muela SH, Ribera JM, Tanner M (1998). Fake malaria and hidden parasites-the ambiguity of malaria. Anthropol Med..

[24] Kamat VR (2006). “I thought it was only ordinary fever!” cultural knowledge and the micropolitics of therapy seeking for childhood febrile illness in Tanzania. Soc. Sci. Med..

[25] Plummer ML, Mshana G, Wight D, Wamoyi J, Salamba Z, Hayes RJ, Ross DA (2006). ‘The man who believed he had AIDS was cured’: AIDS and sexually-transmitted infection treatment-seeking behaviour in rural Mwanza, Tanzania. AIDS Care..

[26] Levi-Strauss C (1963). Structural anthropology. Translated by Claire Jacobson and Brooke Grundfest Schoepf.

[27] Bourdieu P (1989). Social space and symbolic power. Sociological Theory..

[28] Kapiga S, Harvey S, Mshana G, Hansen CH, Mtolela GJ, Madaha F, Hashim R, Kapinga I, Mosha N, Abramsky T, Lees S, Watts C (2019). A social empowerment intervention to prevent intimate partner violence against women in a microfinance scheme in Tanzania: findings from the MAISHA cluster randomised controlled trial. Lancet Glob Health..

[29] Mutambara J, Sodi T, Mtemeri J, Makomo M. Harmonizing religion and health: an exploration of religious reasons for defaulting ARVs among people living with HIV and AIDS in Gweru, Zimbabwe. AIDS Care. 2020; Feb 7;1-6, doi: 10.1080/09540121.2020.1724255. Online ahead of print 10.1080/09540121.2020.1724255 32030992

[30] Isaac JC (1992). “Beyond the free faces of power: a realist critique” in *Rethinking Power*.

[31] Scott J (2001). Power.

[32] Qiao S, Ingram L, Deal ML, Li X, Weissman SB. Resilience resources among African American women living with HIV in Southern United States. AIDS. 2019; 33 Suppl 1, S35-S44, doi: 10.1097/QAD.0000000000002179. 10.1097/QAD.0000000000002179 31397721

[33] Zarzycka B, Śliwak J, Dariusz K, D Paweł C. Religious comfort and anxiety in women with cancer: the mediating role of hope and moderating role of religious struggle. Psycho-Oncology, 2019; 28(9), 1829-1835, doi: 10.1002/pon.5155. Epub 2019 Jul 10. 10.1002/pon.5155 31218773

